# Risk factors of poor outcomes in ANCA-associated vasculitis: a single-center study

**DOI:** 10.1080/0886022X.2025.2519818

**Published:** 2025-07-14

**Authors:** Axi Age, Ruihong Huang, Yinan Li, Juan Pei

**Affiliations:** ^a^ Department of Nephrology, The First Affiliated Hospital of Xiamen University, School of Medicine, Xiamen University, Xiamen, China; ^b^ The School of Clinical Medicine, Fujian Medical University, Fuzhou, China

**Keywords:** ANCA, BUN, dialysis, ESKD, vasculitis, mortality

## Abstract

**Objective:**

Anti-neutrophil cytoplasmic antibody (ANCA)-associated vasculitis (AAV) is a rare disease with high rate of end-stage kidney disease (ESKD) and mortality. We aimed to investigate the risk factors for ESKD and/or death in AAV patients.

**Methods:**

Seventy-eight AAV patients diagnosed from November 2013 to December 2022 were included. Study endpoints included ESKD or death and composite endpoints. Cox regression models were used to adjust for potential baseline confounders.

**Results:**

Among the 78 cases, there were 16 cases of ESKD, 15 cases of death, and 27 cases of composite endpoint during follow-up. After adjusting for baseline characteristics, baseline blood urea nitrogen (BUN) level (adjusted hazard ratio [HR] 1.07, 95%Cl 1.04–1.10) was an independent risk factor for composite endpoints (ESKD and/or death) in AAV patients. ROC curve analysis showed that the optimal cutoff of BUN level was 19.5 mmol/L for ESKD and death (AUC: 0.760; 95% CI: 0.65–0.87). Baseline BUN levels (HR per 1 mmol/L: 1.09, 95% CI 1.05–1.23) and white blood cell (WBC) counts (HR per 1,000/μL: 1.22, 95% CI 1.07–1.40) were independent risk factors for ESKD in AAV. Cardiovascular involvement (HR 8.74, 95%Cl 2.20–34.73), NLR (HR 1.04, 95%Cl 1.01–1.08), and antiplatelet drug use (HR 6.79, 95%Cl 1.33–34.62) were independent risk factors for death in AAV.

**Conclusions:**

These findings suggest that high baseline BUN level was an independent predictor for ESKD and/or death in AAV patients, even after correction for co-founders including Scr. BUN might constitute an independent, easily available and important parameter for risk stratification in AAV.

## Introduction

Anti-neutrophil cytoplasmic antibodies (ANCA)-associated vasculitis (AAV) is a type of systemic autoimmune disease characterized by the presence of ANCA in circulation as an auto-antibody and necrotizing inflammation of small vessel walls. AAV mainly includes the following categories: microscopic polyangiitis (MPA), granulomatosis with polyangiitis (GPA), and eosinophilic granulomatosis polyangiitis (EGPA, formerly known as Churg-Strauss syndrome) [1]. ANCA is typically detected using two main methods: indirect immunofluorescence (IIF) and enzyme-linked immunosorbent assay (ELISA). IIF classifies ANCA into two patterns: cytoplasmic (c-ANCA) and perinuclear (p-ANCA). However, IIF alone does not identify the specific target antigen. ELISA is required to confirm the specific antigens, which are most commonly proteinase 3 (PR3) for c-ANCA and myeloperoxidase (MPO) for p-ANCA [2–4]. In general, combined C-ANCA-PR3 was dominant in patients with GPA (65%), while combined P-ANCA-MPO was dominant in MPA (60%) [5]. Patients with MPA and with positivity for MPO-ANCA are predominant in Asian countries, whereas patients with GPA and with positivity for PR3-ANCA are predominant in northern Europe and the United States [6].

AAV can occur at any age, and the clinical manifestations are diverse and overlapping, depending on disease stage, the specific organ system involvement(s), disease activity/severity and the chronicity/extent of damage to organ system involved. The specific organ involvement varies depending on the clinical phenotype, with MPA primarily affecting the kidneys and lungs [7], GPA often involving the upper respiratory tract and kidneys [8], and EGPA commonly affecting the lungs, nerves, and heart [9]. Kidneys as the highly vascularized organs are affected as high as 90% in MPA and 80% in GPA [10]. Kidney AAV typically presents as rapidly progressive glomerulonephritis (RPGN) which can lead to ESKD in a very short time and contribute to death if not properly treated. Death by AAV mainly occurs within 1 year and the death rate without treatment were higher than 80% [11,12]. With the widespread use of immunosuppressants of AAV patients, the 1-year mortality rate has significantly decreased to approximately 10%-15% [13–16]. While the risk of death among AAV remains high, which was about 2.7 times higher than those of the average population [13].

Several studies have been reported on the predictors of poor outcomes of AAV, while due to the heterogeneity of clinical manifestations and outcomes, this study is intended to explore the potential risk factors of ESKD and/or death in a single center in Chinese AAV patients.

## Patients and methods

### Study population

A total of 78 patients with AAV who were first diagnosed and hospitalized in the First Affiliated Hospital of Xiamen University from November 15, 2013 to December 31, 2022 were included in this retrospective study. Patients included met the following criteria (10): (1) positive ANCA confirmation and screening tests; (2) Complete clinical data; (3) In line with the latest classification and naming consensus of vasculitis published at the Chapill Hill Conference. Patients were excluded from the study for the following reasons: (1) secondary vasculitis due to clear etiology, such as infection, tumor, diffuse connective tissue disease, drugs, while patients with preexisting tumors unrelated to vasculitis pathogenesis were not excluded.; (2) combined with other autoimmune diseases, such as rheumatoid arthritis, systemic lupus erythematosus, and Sjogren’s syndrome; (3) the ANCA screening test was negative; (4) patients with incomplete clinical data. The study has been approved by the local ethics committee of the First Affiliated Hospital of Xiamen University and waived the requirement for written consent.

### Data collection

The following data are collected at the initial diagnosis and treatment of AAV patients: age, sex, organ involvement (skin mucosa, chest, ear-throat-nose, cardiovascular, digestive, renal, eye, nervous system), laboratory data including routine blood and urine tests, liver and kidney function, inflammatory markers, immunoglobulins, complements, ANCA serology, and chest imaging. Indirect immunofluorescence (IIF) is used to detect cytoplasmic and perinuclear antibodies (c-ANCA and p-ANCA). Immunoblotting is used to detect anti-MPO/PR3 antibodies. Renal pathology, when performed, renal biopsies were rated according to Berden et al.’s prognostic classification [17]. It is assessed whether high-dose glucocorticoids (defined as a maximum daily dose of methylprednisolone ≥200mg for 3 consecutive days, followed by oral prednisone tapered gradually), Cytoxan (cyclophosphamide), intravenous immunoglobulin, rituximab, plasma exchange, mechanical ventilation, antiplatelet drugs (clopidogrel, aspirin, dipyridamole), and statins were used during the first hospitalization. Routine and immunological examinations are completed at the Department of Laboratory Medicine, The First Affiliated Hospital of Xiamen University.

### Outcomes

The primary outcome was a composite endpoint of either end stage kidney disease (ESKD, defined as the beginning of long-term dialysis) or death. Secondary outcomes included ESKD and death separately. The record of death and ESKD were obtained either through medical records or telephone follow-ups. Data were censored as of 31st December 2022.

### Statistical analysis

Data are expressed as number (percentage) for categorical variables, mean ± standard deviation for normally distributed continuous variables, and median (interquartile range) for continuous variables that were not normally distributed. Pearson Chi-square or Fisher’s exact test was used for comparison between the two groups for categorical variables. Student’s t-test was performed for normally distributed continuous variables, and Mann–Whitney test or Kruskal–Wallis test for continuous variables that were not normally distributed. Survival analysis was performed using a Cox proportional hazards model. Variables that achieved statistical significance in the univariate analysis were subsequently included in a multivariate analysis using a stepwise Cox regression procedure. Kaplan–Meier cumulative survival curves were constructed and compared using the log rank test. Receiver-operating characteristic (ROC) curves for ESKD and/or death were calculated for each predictor using cutoff values. SPSS25.0 software and Stata/SE14.0 (College Station, TX) were used for analysis. All tests were two-sided, *P* values < 0.05 were considered statistically significant.

## Results

### Study population and baseline characteristics

During the study period, 78 patients who met the inclusion criteria were included. The demographic characteristic of the study population is shown in Table 1.

**Table 1.** Characteristics of study participants (*N* = 78).

Variable	Result
Number of patients	78
Number of males (%)	40 (51.3)
Age (years)	62.50 (51.75, 69.25)
Renal biopsy (﹪)	31 (39.7)
Focal	10 (32.3)
Crescentic	12 (38.7)
Mixed	7 (22.6)
Sclerotic	2 (6.5)
ANCA type	
MPO-ANCA, (﹪)	66 (84.6)
PR3-ANCA, (﹪)	7 (9)
C-ANCA, (﹪)	12 (15.4)
P-ANCA, (﹪)	67 (85.9)
Clinical data	
WBC,10^9^/L	9.13 (6.47, 11.74)
NLR	5.95 (3.04, 8.37)
Hemoglobin,g/L	90.81 ± 21.31
Platelets,10^9^/L	244.50 (196.50, 370.75)
ESR,mm/h	73.62 ± 32.59
Albumin,g/L	32.19 ± 6.23
BUN,mmol/L	13.13 (6.83, 26.06)
Scr,μmol/L	270.00 (123.75, 551.50)
UA,μmol/L	426.23 ± 160.57
IgG,g/L	15.08 ± 4.85
IgA,g/L	2.45 (1.99, 3.19)
IgM,g/L	0.90 (0.65, 1.29)
C3,g/L	0.98 ± 0.26
C4,g/L	0.26 (0.19, 0.32)
Hematuria (red cell counts), n/μl	146.95 (31.78, 470.48)
24h-UTP, g/24 h	0.97 (0.48, 2.40)
Involved organs	
Ear-throat-nose (﹪)	16 (21.0)
Chest (﹪)	27 (34.6)
Kidney (﹪)	73 (93.6)
Digestive (﹪)	28 (35.9)
Cardiovascular (﹪)	9 (11.5)
Musculoskeletal (﹪)	9 (11.5)
Eye (﹪)	5 (6.4)
Nervous system (﹪)	2 (2.6)
Treatment	
Glucocorticoid pulse therapy (﹪)	30 (38.5)
Cytoxan (﹪)	58 (74.4)
Gamma globulin (﹪)	27 (34.6)
Rituximab (﹪)	5 (6.4)
Plasma exchange (﹪)6.4)	11 (14.7)
Mechanical ventilation (﹪)	3 (3.8)
ICU treatment, (﹪)	7 (9.0)
Antiplatelet drugs (﹪)	5 (5.1)
Statin (﹪)	7 (9.0)
Minimal Therapy	19 (24.4)
Standard induction therapy	32 (41.0)
Intensive Therapy	27 (34.6)
Baseline comorbidities	
History of:	
Hypertension, (﹪)	41 (52.6)
Diabetes, (﹪)	14 (18.0)
Tumor, (﹪)	5 (6.4)

ANCA: Anti-Neutrophil Cytoplasmic Antibodies; ICU: intensive care unit. Standard induction therapy: Oral/pulse glucocorticoids + Cytoxan or rituximab). Intensive Therapy: Standard induction therapy + adjunctive interventions (plasma exchange, gamma globulin). Minimal Therapy: Glucocorticoids alone or with ≤1 adjunct. Results are presented as medians (interquartile range) or mean ± standard or frequency (percentage).

There were 40 (51.3%) male and 38 (39.7%) female patients. The median age of onset was 62.50 (51.75, 69.25) years; 16 patients received long-term renal replacement therapy (2 patients with peritoneal dialysis, 14 patients with hemodialysis); 15 patients died (6 pulmonary infection, three heart failure, four vasculitis activity defined by progressive multi-organ failure despite maximal immunosuppression, two unknown reason due to the absence of autopsy and detailed medical records). Only four deaths (26.7% of total deaths) occurred in patients with ESKD.

The majority of AAV patients were MPO-ANCA positive (66 cases, 84.6%). The most commonly involved organs were kidney in 73 cases (93.6%), followed by digestive, respiratory, and cardiovascular systems in 28 cases (35.9%), 27 cases (34.6%), and nine cases (11.5%), respectively.

The most common baseline comorbidities were hypertension in 41 cases (52.6%), diabetes in 14 cases (18.0%) and tumor in 5 cases (6.4%). Among the 6.4% of patients with tumors in our cohort, all had solid tumors (colon cancer = 2, cervical cancer = 1, thymic carcinoma = 1, teratoma = 1) diagnosed more than 5 years prior to vasculitis onset, with long time remission and no evidence of recurrence based on imaging and tumor markers, these patients were retained as their malignancies were unrelated to vasculitis pathogenesis.

In this cohort, only 39.7% of patients (31/78) underwent renal biopsy due to bleeding or life-threatening condition or patient refusal (10 focal, 12 crescentic, 7 mixed, 2 sclerotic).

Glucocorticoids were used in all patients, including 30 patients (38.5%) receiving glucocorticoid pulse therapy for the first time, 48 patients (61.5%) receiving oral prednisone at a dose of 0.5-1 mg/kg/day, which was gradually tapered. 58 patients (74.4%) receiving cyclophosphamide (intravenous 0.5–1 g/m^2^/month), 27 patients (34.6%) receiving gamma globulin, and 5 patients (6.4%) receiving rituximab. Plasma exchange was used in 11 cases (14.7%) and mechanical ventilation in 3 cases (3.8%). 7 patients (9%) receiving statin, and 5 (5.1%) patients using antiplatelet drugs with cardiovascular comorbidities or thrombotic risk. Among them, 32 patients receiving standard induction therapy (SIT) (Oral/pulse glucocorticoids + cyclophosphamide or rituximab), prioritizing regimens with clinical guidelines support; 27 patients receiving intensive therapy (IT): SIT + adjunctive interventions (plasma exchange, gamma globulin). Of the cohort, 19 patients receiving Minimal Therapy (MT): Glucocorticoids alone or with ≤1 adjunct.

### Primary outcome: ESKD-free survival

The total follow-up period of the cohort was 209 patient-years with a median follow-up period of 1.8 years (interquartile range 0.5–4.4 years). There were 27 (34.6%) patients who developed ESKD and/or death during the follow-up period. The incidence of first occurrence of ESKD and/or death was 13.0 events per 100 person-years. The 1-year, 3-year, and 5-year ESKD-free survival rate were 79.1%, 73.7%, and 59.2%, respectively.

The univariate analysis of the relative risk for ESKD and/or death is shown in Tables 2. Neutrophil-to-lymphocyte ratio (NLR) (HR = 1.025, 95%CI:1.007 – 1.043, *p* = 0.005), hemoglobin (HR = 0.970, 95﹪CI: 0.951 – 0.989,*p* = 0.002), blood urea nitrogen (BUN) (HR = 1.068, 95﹪CI:1.041 – 1.095, *p* < 0.001), serum creatinine (Scr) (HR = 1.002, 95﹪CI:1.001 – 1.003, *p* < 0.001), uric acid (UA) (HR = 1.004, 95﹪CI:1.002 – 1.007, *p* < 0.001) were associated with ESKD or death in AAV patients

**Table 2.** Univariate Cox regression analysis of exposure factors related to ESKD and/or death.

Variable	*P* value	HR	95.0% CI	
Number of males (%)	0.991	0.995	0.465	2.129
Age (years)	0.442	1.011	0.983	1.040
Renal biopsy (﹪)	0.450	0.725	0.314	1.671
MPO-ANCA, (﹪)	0.122	3.124	0.737	13.233
PR3-ANCA, (﹪)	0.207	0.040	0.000	5.945
C-ANCA, (﹪)	0.643	0.752	0.225	2.514
P-ANCA, (﹪)	0.629	1.345	0.404	4.482
WBC,10^9^/L	0.067	1.077	0.995	1.167
NLR	0.005	1.025	1.007	1.043
Hemoglobin,g/L	0.002	0.970	0.951	0.989
Platelets,10^9^/L	0.773	1.000	0.997	1.003
ESR,mm/h	0.680	1.003	0.989	1.017
Albumin,g/L	0.410	0.974	0.913	1.038
BUN,mmol/L	<0.001	1.068	1.041	1.095
Scr,μmol/L	<0.001	1.002	1.001	1.003
UA,μmol/L	0.001	1.004	1.002	1.007
IgG,g/L	0.406	0.961	0.875	1.056
IgA,g/L	0.394	0.835	0.551	1.265
IgM,g/L	0.817	1.103	0.480	2.538
C3,g/L	0.936	0.938	0.199	4.424
C4,g/L	0.079	1.626	0.944	2.799
Hematuria (red cell counts), n/μL	0.464	1.000	0.999	1.001
24h-UTP, g/24 h	0.490	1.077	0.873	1.329
Ear-throat-nose (﹪)	0.486	1.345	0.585	3.093
Chest (﹪)	0.762	0.886	0.404	1.944
Kidney (﹪)	0.298	23.704	0.061	9166.237
Digestive (﹪)	0.090	1.938	0.903	4.158
Cardiovascular (﹪)	0.071	2.503	0.924	6.778
Musculoskeletal (﹪)	0.226	0.290	0.039	2.149
Eye (﹪)	0.408	0.429	0.058	3.190
Nervous system (﹪)	0.722	1.441	0.192	10.817
Interstitial lung disease, (﹪)	0.636	1.209	0.551	2.655
Pleural effusion, (﹪)	0.632	1.218	0.543	2.735
Lung infiltration, (﹪)	0.529	1.281	0.593	2.767
Lung nodules, (﹪)	0.863	0.917	0.344	2.444
Emphysema, (﹪)	0.853	0.904	0.311	2.625
Glucocorticoid pulse therapy (﹪)	0.704	1.163	0.534	2.532
Cyclophosphamide (Cytoxan) (﹪)	0.209	1.803	0.719	4.519
Gamma globulin (﹪)	0.728	1.148	0.527	2.500
Rituximab (﹪)	0.509	0.045	0.000	443.535
Plasma exchange (﹪)6.4)	0.855	1.105	0.381	3.205
Minimal Therapy		Ref		
Standard induction therapy	0.337	1.660	0.590	4.672
Intensive Therapy	0.244	1.814	0.666	4.943
Mechanical ventilation (﹪)	0.376	0.043	0.000	45.503
ICU treatment, (﹪)	0.933	0.940	0.221	3.994
Antiplatelet drugs (﹪)	0.472	1.702	0.400	7.247
Statin (﹪)	0.637	1.426	0.327	6.207
Hypertension, (﹪)	0.179	1.715	0.780	3.767
Diabetes, (﹪)	0.246	1.667	0.703	3.954
Tumor, (﹪)	0.649	1.399	0.329	5.943

ESKD: end stage kidney disease; ANCA: Anti-Neutrophil Cytoplasmic Antibodies; WBC: white blood cell; NLR: Neutrophil-to-lymphocyte ratio; ESR: erythrocyte sedimentation rate; BUN: blood urea nitrogen; Scr: serum creatinine; UA: uric acid; IgG: immunoglobulin G; IgA: immunoglobulin A; IgM: immunoglobulin M; C3: complement component 3; C4: complement component 4; 24h-UTP: 24h urine total protein quantity; ICU: intensive care unit. Standard induction therapy: Oral/pulse glucocorticoids + Cytoxan or rituximab). Intensive Therapy: Standard induction therapy + adjunctive interventions (plasma exchange, gamma globulin). Minimal Therapy: Glucocorticoids alone or with ≤1 adjunct.

**Figure 1.**
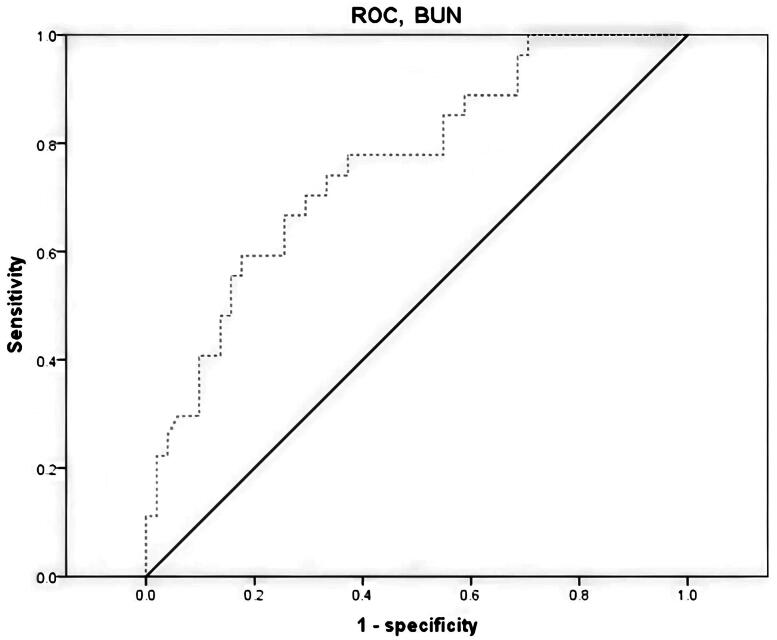
The ROC curve of BUN as a predictor of ESKD and/or death in patients with AAV.

**Figure 2.**
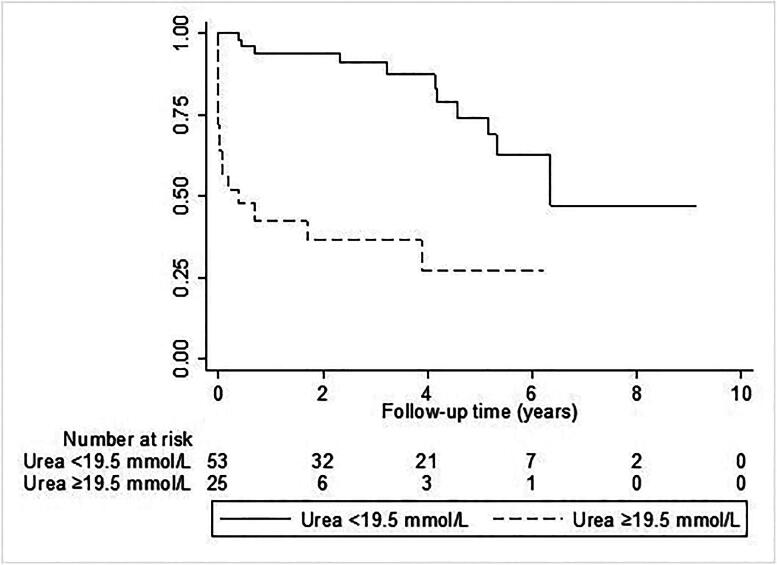
An admission BUN concentration above 19.5 mg/dL, the optimal cutoff calculated by Youden index, is associated with ESKD and/or death, depicted as Kaplan-Meier curve, group comparison by log-rank test, p-value <0.001.

Multivariate Cox regression analysis showed that only BUN (HR = 1.065, 95﹪CI:1.039 – 1.093, *p* < 0.001) was an independent risk factor for ESKD-free survival in AAV patients (Table 3). There was no evidence of significant two-way interaction terms. We performed ROC-analysis for BUN (AUC 0.76 95%CI: 0.65–0.87, *p* < 0.001, Figure 1). Further, an optimal admission BUN cutoff was calculated at 19.5 mg/dL. This cutoff was associated with ESKD-free survival (HR 6.91; 95%CI: 3.06–15.57; *p* < 0.001, Figure 2).

**Table 3.** Multivariate cox regression analysis of risk factors of ESKD and/or death.

Variable	*P* value	HR	95 % CI	
BUN	<0.001	1.065	1.039	1.093

ESKD: end-stage kidney disease; BUN: blood urea nitrogen.

### Kidney survival

The total follow-up period of the cohort was 209 patient-years with a median follow-up period of 1.8 years (interquartile range 0.5–4.4 years). Within the entire cohort, there were 16 (20.5%) patients who developed ESKD during the follow-up period. The incidence of first occurrence of ESKD was 7.7 events per 100 person-years. The 1-year, 3-year, and 5-year ESKD-free survival rate were 84.0%, 78.3%, and 74.9%, respectively.

The univariate analysis of the relative risk for ESKD is shown in Table 4. White blood cell count (WBC, HR = 1.141, 95%CI: 1.026 – 1.268, *p* = 0.015), hemoglobin (HR = 0.969, 95%CI: 0.946 – 0.993,*p* = 0.010), BUN (HR = 1.077, 95%CI: 1.045 – 1.111, *p* < 0.001), Scr (HR = 1.002, 95%CI: 1.001 – 1.003, *p* < 0.001), uric acid (HR = 1.005, 95%CI:1.002 – 1.008, *p* = 0.003), C4 (HR = 1.837, 95%CI:1.096 – 3.081, *p* = 0.021), digestive system (HR = 3.335, 95%CI: 1.183 – 9.398, *p* = 0.023) was associated with ESKD in ANCA-associated vasculitis patients.

**Table 4.** Univariate Cox regression analysis of exposure factors related to death.

Variable	*P* value	HR	95.0% CI	
Number of males (%)	0.198	2.077	0.682	6.322
Age (years)	0.038	1.054	1.003	1.109
Renal biopsy (﹪)	0.094	0.273	0.059	1.250
Renal replacement (PD or HD),﹪	0.379	1.683	0.528	5.364
MPO-ANCA,﹪	0.649	1.417	0.315	6.369
PR3-ANCA,﹪	0.367	0.040	0.000	43.300
C-ANCA,﹪	0.722	1.318	0.289	6.020
P-ANCA,﹪	0.864	0.876	0.194	3.956
WBC,10^9^/L	0.548	1.036	0.924	1.162
NLR	0.005	1.025	1.007	1.043
Hemoglobin,g/L	0.146	0.980	0.953	1.007
Platelets,10^9^/L	0.627	0.999	0.995	1.003
ESR,mm/h	0.968	1.000	0.982	1.019
Albumin,g/L	0.155	0.937	0.857	1.025
BUN,mmol/L	0.042	1.037	1.001	1.075
Scr,μmol/L	0.100	1.001	1.000	1.002
UA,μmol/L	0.167	1.002	0.999	1.005
IgG,g/L	0.611	0.968	0.854	1.097
IgA,g/L	0.631	1.139	0.669	1.940
IgM,g/L	0.974	0.980	0.295	3.259
C3,g/L	0.871	1.191	0.145	9.779
C4,g/L	0.055	1.771	0.987	3.178
Hematuria (red cell counts), n/μL	0.989	1.000	0.999	1.001
24h-UTP, g/24 h	0.632	1.079	0.790	1.473
Ear-throat-nose (﹪)	0.745	1.213	0.378	3.890
Chest (﹪)	0.147	2.169	0.762	6.177
Kidney (﹪)	0.475	23.396	0.004	132298.660
Digestive (﹪)	0.896	0.926	0.291	2.943
Cardiovascular (﹪)	<0.001	10.020	2.891	34.729
Musculoskeletal (﹪)	0.743	0.709	0.091	5.511
Eye (﹪)	0.908	0.886	0.114	6.877
Nervous system(﹪)	0.543	0.043	0.000	1063.662
Interstitial lung disease,(﹪)	0.356	1.652	0.568	4.802
Pleural effusion,(﹪)	0.178	2.119	0.710	6.324
Lung infiltration,(﹪)	0.131	2.236	0.787	6.353
Lung nodules,(﹪)	0.751	0.782	0.171	3.579
Emphysema,(﹪)	0.959	1.041	0.230	4.704
Glucocorticoid pulse therapy (﹪)	0.334	1.682	0.586	4.831
Cyclophosphamide (Cytoxan) (﹪)	0.196	2.358	0.642	8.659
Gamma globulin (﹪)	0.992	1.005	0.355	2.851
Rituximab (﹪)	0.717	0.046	0.000	756781.948
Plasma exchange (﹪)6.4)	0.418	1.698	0.472	6.110
Minimal Therapy		Ref		
Standard induction therapy	0.230	2.494	0.561	11.081
Intensive Therapy	0.271	2.193	0.542	8.868
Mechanical ventilation (﹪)	0.517	0.043	0.000	581.624
ICU treatment,(﹪)	0.254	2.418	0.531	11.014
Antiplatelet drugs (﹪)	0.041	4.955	1.066	23.032
Statin (﹪)	0.433	2.363	0.276	20.255
Hypertension,(﹪)	0.547	1.376	0.487	3.886
Diabetes, (﹪)	0.811	1.157	0.349	3.838
Tumor, (﹪)	0.080	3.899	0.849	17.909

ANCA: Anti-Neutrophil Cytoplasmic Antibodies; WBC: white blood cell; NLR: Neutrophil-to-lymphocyte ratio; ESR: erythrocyte sedimentation rate; BUN: blood urea nitrogen; Scr: serum creatinine; UA: uric acid; IgG: immunoglobulin G; IgA: immunoglobulin A; IgM: immunoglobulin M; C3: complement component 3; C4: complement component 4; 24h-UTP: 24h urine total protein quantity; ICU: intensive care unit. Standard induction therapy: Oral/pulse glucocorticoids + Cytoxan or rituximab). Intensive Therapy: Standard induction therapy + adjunctive interventions (plasma exchange, gamma globulin). Minimal Therapy: Glucocorticoids alone or with ≤1 adjunct.

Multivariate Cox regression analysis showed that the WBC (HR = 1.224, 95%CI: 1.069 – 1.403, *p* = 0.004) and BUN (HR = 1.091, 95%CI: 1.054 - 1.129, *p* < 0.001) were independent risk factors for ESKD in ANCA-associated vasculitis. (Table 5). There was no evidence of significant two-way interaction terms.

**Table 5.** Multivariate Cox regression analysis of risk factors of death.

Variable	*P* value	HR	95.0% CI	
NLR	0.009	1.042	1.010	1.075
Cardiovascular	0.002	8.740	2.200	34.725
Antiplatelet drugs	0.021	6.789	1.331	34.623

NLR: Neutrophil-to-lymphocyte ratio.

### Patient survival

The total follow-up period of the cohort was 241 patient-years with a median follow-up period of 2.8 years (interquartile range 1.2–4.6 years). There were 15 (19.2%) patients who developed death during the follow-up period. The incidence of first occurrence of death was 6.2 events per 100 person-years. Overall, the 1-year survival rate was 92.0%, 3-year rate was the same and the 5-year rate was 76.1%.

The univariate analysis of the relative risk for death is shown in Tables 6. Age (HR = 1.054, 95%CI:1.003 – 1.109, *p* = 0.038), NLR (HR = 1.025, 95%CI:1.007 – 1.043, *p* = 0.005), blood urea nitrogen (HR = 1.037, 95%CI:1.001 – 1.075, *p* = 0.042), cardiovascular system (HR= 10.020, 95%CI: 2.891 – 34.729, *p* < 0.001), coronary heart disease (HR = 4.635, 95%CI:1.244 – 17.267, *p* = 0.022) and antiplatelet drugs (HR = 4.955, 95%CI: 1.066-23.032, *p* = 0.041) were associated with death in AAV patients

**Table 6.** Univariate Cox regression analysis of exposure factors related to ESKD.

Variable	*P* value	HR	95% CI	
Number of males (%)	0.309	1.709	0.608	4.803
Age (years)	0.356	0.985	0.955	1.017
Renal biopsy(﹪)	0.548	1.367	0.493	3.788
MPO-ANCA,(﹪)	0.244	28.020	0.103	7611.808
PR3-ANCA,(﹪)	0.373	0.041	0.000	45.828
C-ANCA,(﹪)	0.763	0.795	0.179	3.529
P-ANCA,(﹪)	0.853	1.151	0.260	5.106
WBC,10^9^/L	0.015	1.141	1.026	1.268
NLR	0.439	1.013	0.981	1.046
Hemoglobin,g/L	0.010	0.969	0.946	0.993
Platelets,10^9^/L	0.486	1.001	0.998	1.005
ESR,mm/h	0.848	1.002	0.984	1.019
Albumin,g/L	0.659	0.982	0.904	1.066
BUN,mmol/L	<0.001	1.077	1.045	1.111
Scr,μmol/L	<0.001	1.002	1.001	1.003
UA,umol/L	0.003	1.005	1.002	1.008
IgG,g/L	0.593	0.968	0.857	1.092
IgA,g/L	0.131	0.620	0.334	1.153
IgM,g/L	0.566	1.359	0.477	3.872
C3,g/L	0.761	1.398	0.162	12.095
C4,g/L	0.021	1.837	1.096	3.081
Hematuria (red cell counts), n/μL	0.193	1.001	0.999	1.001
24h-UTP, g/24 h	0.314	1.125	0.895	1.414
Ear-throat-nose (﹪)	0.682	1.271	0.403	4.013
Chest(﹪)	0.160	0.403	0.114	1.433
Kidney(﹪)	0.023	3.335	1.183	9.398
Digestive(﹪)	0.449	23.075	0.007	77581.847
Cardiovascular(﹪)	0.660	0.633	0.083	4.835
Musculoskeletal (﹪)	0.348	0.041	0.000	32.821
Eye(﹪)	0.476	0.044	0.000	236.092
Nervous system(﹪)	0.243	3.365	0.438	25.841
Interstitial lung disease,(﹪)	0.731	0.818	0.260	2.572
Pleural effusion,(﹪)	0.841	1.116	0.381	3.265
Lung infiltration,(﹪)	0.703	0.818	0.291	2.299
Lung nodules,(﹪)	0.635	1.321	0.419	4.168
Emphysema,(﹪)	0.779	1.199	0.338	4.250
Glucocorticoid pulse therapy (﹪)	0.994	1.004	0.357	2.823
Cyclophosphamide (Cytoxan) (﹪)	0.394	1.739	0.487	6.204
Gamma globulin (﹪)	0.364	1.600	0.580	4.416
Rituximab (﹪)	0.581	0.045	0.000	2708.653
Plasma exchange(﹪)	0.993	1.006	0.227	4.464
Minimal Therapy		Ref		
Standard induction therapy	0.548	1.535	0.380	6.198
Intensive Therapy	0.299	2.057	0.528	8.011
Mechanical ventilation(﹪)	0.550	0.046	0.000	1146.069
ICU treatment,(﹪)	0.781	0.749	0.098	5.713
Antiplatelet drugs (﹪)	0.552	0.046	0.000	1180.435
Statin (﹪)	0.959	0.948	0.123	7.296
Hypertension,(﹪)	0.502	1.425	0.507	4.008
Diabetes,(﹪)	0.131	2.286	0.781	6.696
Tumor,(﹪)	0.518	0.045	0.000	538.002

ESKD: end-stage kidney disease; ANCA: Anti-Neutrophil Cytoplasmic Antibodies; WBC: white blood cell; NLR: Neutrophil-to-lymphocyte ratio; ESR: erythrocyte sedimentation rate; BUN: blood urea nitrogen; Scr: serum creatinine; UA: uric acid; IgG: immunoglobulin G; IgA: immunoglobulin A; IgM: immunoglobulin M; C3: complement component 3; C4: complement component 4; 24h-UTP: 24h urine total protein quantity; ICU: intensive care unit. Standard induction therapy: Oral/pulse glucocorticoids + Cytoxan or rituximab). Intensive Therapy: Standard induction therapy + adjunctive interventions (plasma exchange, gamma globulin). Minimal Therapy: Glucocorticoids alone or with ≤1 adjunct.

Multivariate Cox regression analysis showed that the NLR (HR = 1.042, 95%CI: 1.010 – 1.075, *p* = 0.009), cardiovascular diseases (HR = 8.740, 95% CI: 2.200 – 34.725, *p* = 0.002), antiplatelet agents (HR = 6.789, 95% CI:1.331 – 34.623, *p* = 0.021) were an independent risk factor for mortality in AAV patients (Table 7). There was no evidence of significant two-way interaction terms.

**Table 7.** Multivariate Cox regression analysis of risk factors of ESKD.

Variable	*P* value	HR	95.0% CI	
WBC	0.004	1.224	1.069	1.403
BUN	<0.001	1.091	1.054	1.129

ESKD: end-stage kidney disease; WBC: white blood cell; BUN: blood urea nitrogen.

## Discussion

In this retrospective study, we examined the risk factors of ESKD or death in 78 AAV patients with a median follow-up period of 2.8 years. BUN levels were the only risk factor associated with this combined poor outcome, and this association remained statistically significant even after the adjustment for cofounding factors in multivariable modeling. BUN level ≥19.5 mmol/L was an independent risk factor for that composite outcome, which suggests elevated BUN levels (≥19.5 mmol/L) may serve as a convenient indicator of the severity and poor prognosis in AAV patients. BUN and WBC at baseline were independent risk factors for ESKD. Furthermore, cardiovascular involvement at initial diagnosis, treatment with antiplatelet agents, and high NLR were also independent risk factors for death.

Many studies have examined predictors of outcome in AAV, there are only a few studies looking at the predictors of the composite outcome (ESKD and/or death). As the prognosis of AAV improved after the use of immunosuppressive drugs, it is also vital to find the risk factors of survival without dialysis in AAV. A Southeastern United States study showed that the 1-year and 5-year ESKD-free survival rate was 75% and 54% [14]. In the present study, the 1-year, 3-year and 5-year ESKD-free survival rates were 79%, 74% and 59%, which was in line with their cohort. In their study, 554 patients were analyzed with a median follow-up of 31 months showed that Scr levels were the only risk factor of ESKD-free survival ^[^14]. BUN was not included as a potential exposure factor in this study might be the one cause of the differences. In our study, BUN was remain strongly associated with ESKD-free survival even after controlling of Scr.

To evaluate whether covariates modified the association between BUN and ESKD-free survival, we tested two-way interaction between BUN and all covariates in the final multivariable Cox model. No statistically significant interactions were detected. Although not all the variables confounding can be controlled in this study, the results showed that BUN have important prognostic implications beyond that contributed by the known confounders.

In line with previous studies, our study also showed that BUN was a strong predictor of ESKD in AAV patients [18,19]. Kawai et al. [18]. found in a study that BUN, Scr and hemoglobin, LDH, proteinuria at the baseline were significant risk factors for the development of ESKD after age- and sex-adjustments. Yoo BW et al. [19] also pointed out that through multivariate Cox regression analysis, BUN, Scr and serum albumin examined at the first hospitalization were significant and were predictive factors of ESKD occurrence. While in contrast to other studies [20], Scr was excluded after the multivariable analysis, which shown an additive value of BUN to Scr in AAV patients.

Notably, BUN demonstrated superior predictive value over Scr for ESKD and ESKD-free survival in our cohort, underscoring pathophysiological distinctions between these biomarkers. As we all known, both BUN and Scr are filtered from glomerulus and considered as the reflection of renal function. While BUN also affected by other factors, such as protein intake, corticosteroids, gastrointestinal hemorrhage or presence volume depletion, among others. The severity of any metabolic disarray that can lead to alterations in urea production, absorption, or excretion, therefore BUN can be considered as a marker of all metabolic activities, neurohumoral activities and renal function as urea reabsorption is altered not only by renal but also any metabolic disarray [21]. This distinction likely stems from BUN’s capacity to integrate multiple pathophysiological processes beyond glomerular filtration rate (GFR) reduction. Specifically, elevated BUN levels reflect not only impaired glomerular filtration but also hypercatabolic states driven by systemic inflammation, and renal hypoperfusion secondary to volume depletion and so on. These superimposed insults collectively indicate a more profound baseline depletion of renal functional reserve and a maladaptive renal microenvironment characterized by intensified tubulointerstitial hypoxia and oxidative stress. Such synergistic deterioration accelerates irreversible nephron loss, ultimately propelling disease progression.

Increased WBC might be associated with infection, which can accelerate kidney function deterioration and leading to ESKD. Therefore, BUN, and WBC at diagnosis could predict ESKD after full adjustment in AAV patients.

In Rhee’s study [14], the 1-year patient survival for the entire cohort was 91% and 5-year survival was 72%. Our findings are consistent with theirs, and the highest yearly incidence of death occurred within the first year. The cardiovascular involvement at initial diagnosis was an independent risk factor for death in AAV patients have been documented by previous study. Heijl et al. [16] found in a population-based cohort study in Swedish that patients with AAV combined with cardiac involvement had a higher mortality rate. Hazebroek et al. [22] also highlighted that the increased mortality in AAV patients with cardiovascular disease. Berti A et al. [23] followed AAV patients for up to 20 years and found that the risk of cardiovascular events in AAV patients was 3 times higher than that in the control group, and the risk of deep vein thrombosis was 6 times higher. The potential mechanism to explain the relationship between cardiovascular system involvement and mortality remain unclear. As a small vessel vasculitis, AAV is characterized by necrotizing inflammation of small vessel walls, which might result in atherosclerosis and vascular sclerosis and contribute to cardiovascular damage [24–26]. Both autoimmune-mediated myocardial inflammation and accelerated atherosclerosis may cause cardiovascular disease in AAV patients and thus subject the patients to high mortality when comorbidity occurs [27]. Further study is warranted in this field. Treatment with antiplatelet agents at initial diagnosis can reflect hypercoagulable state and/or local thrombosis in AAV patients, which might mediate adverse cardiovascular events and mortality. While the limited number of patients on antiplatelet therapy (*n* = 4) precludes robust conclusions. Larger cohorts are needed to disentangle drug effects from baseline risk.

It is interesting to note that our study found that higher NLR at diagnosis can also predict mortality in AAV patients and this observation has been confirmed by Huang et al. [28]. Fonseca et al. [29] found that NLR was an independent predictor of severe infection within 3 months after the onset of immunosuppression at diagnosis and was associated with 1-year mortality. The NLR is a biomarker which combines two different immune pathways, as neutrophil counts are usually elevated to bacterial infection and lymphocyte counts are usually decreased when adaptive immunity suppressed or vital infection, has shown prognostic ability in multiple clinical settings ^[^30–32]. The plausible explanation for the strength of NLR to predict patients’ survival might be that NLR is a more reliable and complementary marker than absolute lymphocyte and neutrophil count separately to reflect the inflammatory load of AAV patients.

Our study highlights a critical dissociation between renal and extra-renal outcomes in AAV: while elevated BUN robustly predicted ESKD, mortality was predominantly driven by non-renal mechanisms (e.g. cardiovascular events, infections). Notably, 11 of 15 deaths (73.3%) occurred in patients without ESKD, underscoring that systemic inflammation (reflected by NLR) and cardiovascular burden—not uremia—were the main drivers of mortality.

Our study has several limitations. First, only a subset of patients underwent renal biopsy; thus, pathological findings were not analyzed in the Cox proportional hazards models, which may introduce potential bias. Second, all patients were from a single center, the sample size was modest, and the follow-up duration was relatively short. Third, the lack of quantitative ANCA titers and standardized organ involvement scoring systems limited our ability to explore dose-response relationships or refine risk stratification. Thus, large-scale multicenter studies incorporating these parameters are needed to validate and extend our findings.

In conclusion, BUN levels at admission were robustly associated with ESKD and ESKD-free survival in our cohort study after correction for several relevant co-founders. Cardiovascular involvement at initial diagnosis, treatment with antiplatelet agents, and NLR were independent predictors for patient survival during follow-up in AAV patients.

## Data Availability

The data underlying this article will be shared on reasonable request to the corresponding author.

## References

[1] Jennette JC. Overview of the 2012 revised International Chapel Hill Consensus Conference nomenclature of vasculitides. Clin Exp Nephrol. 2013;17(5):603–606. doi: 10.1007/s10157-013-0869-6. 24072416 PMC4029362

[2] Falk RJ, Jennette JC. Anti-neutrophil cytoplasmic autoantibodies with specificity for myeloperoxidase in patients with systemic vasculitis and idiopathic necrotizing and crescentic glomerulonephritis. N Engl J Med. 1988;318(25):1651–1657. doi: 10.1056/NEJM198806233182504. 2453802

[3] Jenne DE, Tschopp J, Lüdemann J, et al. Wegener’s autoantigen decoded. Nature. 1990;346(6284):520–520. doi: 10.1038/346520a0. 2377228

[4] Csernok E, Damoiseaux J, Rasmussen N, et al. Evaluation of automated multi-parametric indirect immunofluorescence assays to detect anti-neutrophil cytoplasmic antibodies (ANCA) in granulomatosis with polyangiitis (GPA) and microscopic polyangiitis (MPA). Autoimmun Rev. 2016;15(7):736–741. doi: 10.1016/j.autrev.2016.03.010. 26970486

[5] Geetha D, Jefferson JA. ANCA-associated vasculitis: core curriculum 2020. Am J Kidney Dis. 2020;75(1):124–137. doi: 10.1053/j.ajkd.2019.04.031. 31358311

[6] Katsuyama T, Sada K-E, Makino H. Current concept and epidemiology of systemic vasculitides. Allergol Int. 2014;63(4):505–513. doi: 10.2332/allergolint.14-RAI-0778. 25339434

[7] Binda V, Moroni G, Messa P. ANCA-associated vasculitis with renal involvement. J Nephrol. 2018;31(2):197–208. doi: 10.1007/s40620-017-0412-z. 28560688

[8] Potentas-Policewicz M, Fijolek J. Granulomatosis with polyangiitis: clinical characteristics and updates in diagnosis. Front Med (Lausanne). 2024;11:1369233. doi: 10.3389/fmed.2024.1369233. 39257888 PMC11385631

[9] White JPE, Dubey S. Eosinophilic granulomatosis with polyangiitis: a review. Autoimmun Rev. 2023;22(1):103219. doi: 10.1016/j.autrev.2022.103219. 36283646

[10] Rowaiye OO, Kusztal M, Klinger M. The kidneys and ANCA-associated vasculitis: from pathogenesis to diagnosis. Clin Kidney J. 2015;8(3):343–350. doi: 10.1093/ckj/sfv020. 26034600 PMC4440467

[11] Walton EW. Giant-cell granuloma of the respiratory tract (Wegener’s granulomatosis). Br Med J. 1958;2(5091):265–270. doi: 10.1136/bmj.2.5091.265. 13560836 PMC2026251

[12] Fauci AS, Haynes BF, Katz P, et al. Wegener’s granulomatosis: prospective clinical and therapeutic experience with 85 patients for 21 years. Ann Intern Med. 1983;98(1):76–85. doi: 10.7326/0003-4819-98-1-76. 6336643

[13] Tan JA, Dehghan N, Chen W, et al. Mortality in ANCA-associated vasculitis: ameta-analysis of observational studies. Ann Rheum Dis. 2017;76(9):1566–1574. doi: 10.1136/annrheumdis-2016-210942. 28468793

[14] Rhee RL, Hogan SL, Poulton CJ, et al. Trends in long-term outcomes among patients with antineutrophil cytoplasmic antibody–associated vasculitis with renal disease. Arthritis Rheumatol. 2016;68(7):1711–1720. doi: 10.1002/art.39614. 26814428 PMC4920688

[15] Flossmann O, Berden A, De Groot K, et al. Long-term patient survival in ANCA-associated vasculitis. Ann Rheum Dis. 2011;70(3):488–494. doi: 10.1136/ard.2010.137778. 21109517

[16] Heijl C, Mohammad AJ, Westman K, et al. Long-term patient survival in a Swedish population-based cohort of patients with ANCA-associated vasculitis. RMD Open. 2017;3(1):e000435. doi: 10.1136/rmdopen-2017-000435. 28955485 PMC5604606

[17] Berden AE, Ferrario F, Hagen EC, et al. Histopathologic classification of ANCA-associated glomerulonephritis. J Am Soc Nephrol. 2010;21(10):1628–1636. doi: 10.1681/ASN.2010050477. 20616173

[18] Kawai H, Banno S, Kikuchi S, et al. Retrospective analysis of factors predicting end-stage renal failure or death in patients with microscopic polyangiitis with mainly renal involvement. Clin Exp Nephrol. 2014;18(5):795–802. doi: 10.1007/s10157-013-0926-1. 24363101

[19] Yoo BW, Song JJ, Park YB, et al. Clinical features of Korean elderly patients with antineutrophil cytoplasmic antibody-associated vasculitis. Korean J Intern Med. 2021;36(3):731–741. doi: 10.3904/KJIM.2020.039. 32811130 PMC8137396

[20] Guo Q, Yu L, Zhang X, et al. Analysis of the risk factors for end-stage renal disease and mortality in ANCA-associated vasculitis: a study from a single center of the Chinese Rheumatism Data Center. Clin Rheumatol. 2023;42(2):489–499. doi: 10.1007/s10067-022-06419-1. 36367596

[21] Aronson D, Mittleman MA, Burger AJ. Elevated blood urea nitrogen level as a predictor of mortality in patients admitted for decompensated heart failure. Am J Med. 2004;116(7):466–473. doi: 10.1016/j.amjmed.2003.11.014. 15047036

[22] Hazebroek MR, Kemna MJ, Schalla S, et al. Prevalence and prognostic relevance of cardiac involvement in ANCA-associated vasculitis: eosinophilic granulomatosis with polyangiitis and granulomatosis with polyangiitis. Int J Cardiol. 2015;199:170–179. doi: 10.1016/j.ijcard.2015.06.087. 26209947

[23] Berti A, Matteson EL, Crowson CS, et al. Risk of Cardiovascular Disease and Venous Thromboembolism Among Patients With Incident ANCA-Associated Vasculitis: a 20-Year Population-Based Cohort Study. Mayo Clin Proc. 2018;93(5):597–606. doi: 10.1016/j.mayocp.2018.02.010. 29588079 PMC6057792

[24] Raza K, Thambyrajah J, Townend JN, et al. Suppression of inflammation in primary systemic vasculitis restores vascular endothelial function: lessons for atherosclerotic disease? Circulation. 2000;102(13):1470–1472. doi: 10.1161/01.CIR.102.13.1470. 11004134

[25] Chironi G, Pagnoux C, Simon A, et al. Increased prevalence of subclinical atherosclerosis in patients with small-vessel vasculitis. Heart. 2007;93(1):96–99. doi: 10.1136/hrt.2006.088443. 16940394 PMC1861337

[26] Nienhuis HLA, De Leeuw K, Smit AJ, et al. Enhanced endothelium-dependent microvascular responses in patients with Wegener’s granulomatosis. Journal of Rheumatology. 2007;34(9):1875–1881. 17659753

[27] Knockaert DC. Cardiac involvement in systemic inflammatory diseases. Eur Heart J. 2007;28(15):1797–1804. doi: 10.1093/eurheartj/ehm193. 17562669

[28] Huang L, Shen C, Zhong Y, et al. The association of neutrophil-to-lymphocyte ratio with all-cause mortality in Chinese patients with MPO-ANCA associated vasculitis. Clin Exp Med. 2020;20(3):401–408. doi: 10.1007/s10238-020-00629-0. 32318926

[29] Fonseca JA, Gameiro J, Duarte I, et al. The neutrophil-to-lymphocyte ratio as a marker of vasculitis activity, severe infection and mortality in ANCA-associated vasculitis: a retrospective study. Nefrologia (Engl Ed). 2021;41(3):321–328. doi: 10.1016/j.nefro.2020.07.013. 36165341

[30] Li X, Liu C, Mao Z, et al. Predictive values of neutrophil-to-lymphocyte ratio on disease severity and mortality in COVID-19 patients: a systematic review and meta-analysis. Crit Care. 2020;24(1):647. doi: 10.1186/s13054-020-03374-8. 33198786 PMC7667659

[31] Duchesne JC, Tatum D, Jones G, et al. Multi-institutional analysis of neutrophil-to-lymphocyte ratio (NLR) in patients with severe hemorrhage: a new mortality predictor value. J Trauma Acute Care Surg. 2017;83(5):888–893. doi: 10.1097/TA.0000000000001683. 28837540

[32] Song M, Graubard BI, Rabkin CS, et al. Neutrophil-to-lymphocyte ratio and mortality in the United States general population. Sci Rep. 2021;11(1):464. doi: 10.1038/s41598-020-79431-7. 33431958 PMC7801737

